# Testing Associations Between Childhood Abuse and Health in Young Adults in the Deep South: The Mediating Role of Psychological Symptoms

**DOI:** 10.3390/bs16071127

**Published:** 2026-07-06

**Authors:** Megan E. Renna, Kelsi Broich

**Affiliations:** Department of Psychology, The Ohio State University, 1835 Neil Avenue, Columbus, OH 43210, USA; broich.3@buckeyemail.osu.edu

**Keywords:** childhood trauma, depression, anxiety, sleep, pain, fatigue

## Abstract

**Objective:** Childhood abuse and neglect can have lasting impacts on mental and physical health. These effects may be amplified in rural and/or under-resourced communities, resulting in heightened anxiety, depression, and physical health problems. This study tested associations between childhood abuse/neglect with self-reported physical symptoms, with depressive and anxiety symptoms serving as potential mediators in this association. **Participants:** Participants (N = 606) were undergraduate college students living in the southeastern United States. **Methods:** Participants completed measures assessing childhood abuse/neglect, anxiety and depressive symptoms, and self-rated health, pain, fatigue, and sleep quality. **Results:** Anxiety symptoms mediated the association between emotional, physical, and sexual abuse with all self-reported health outcomes. Depressive symptoms mediated associations between emotional and physical abuse with all health outcomes. **Conclusions:** Results highlight the importance of increasing treatment access among college-aged adults with abuse histories to help mitigate its long-term effects on physical and mental health as individuals age.

## 1. Introduction

The Centers for Disease Control and Prevention (CDC, 2024) identifies childhood abuse as a significant public health concern. Beyond its immediate effects on development during childhood and adolescence, adverse childhood experiences (ACEs)—including abuse and neglect—have enduring consequences for mental health across the lifespan. Research has linked ACEs to increased symptoms of depression, higher rates of suicide attempts, more intensive substance use, and reduced overall well-being in adulthood (Merrick et al., 2017; Nurius et al., 2015). Adverse childhood experiences are also strongly associated with anxiety in adults (Dalechek et al., 2024). Adults with a history of childhood adversity also report experiencing more frequent daily stressors and elevated negative emotional states compared to those without such histories (Mosley-Johnson et al., 2021). The biological embedding hypothesis further explains how early trauma may lead to lasting physiological changes, increasing vulnerability to physical health issues in later life (Danese et al., 2011; G. E. Miller et al., 2011). Within this framework, childhood adversity can become biologically embedded through repeated activation of stress-responsive physiological systems, ultimately producing enduring alterations in neuroendocrine, immune, and autonomic functioning that increase vulnerability to poorer physical health as someone ages. Indeed, childhood trauma has been associated with a range of chronic health conditions in adulthood, such as cardiopulmonary symptoms, chronic pain, obesity, gastrointestinal disorders, and lower self-perceived health (Lovis-Schmidt et al., 2024; Sachs-Ericsson et al., 2005; Wegman & Stetler, 2009).

Independent of chronic conditions, histories of trauma in childhood and adolescence can promote increased physical symptoms among physically healthy adults and those living with a chronic illness. Adults who endured physical abuse as a child are more likely to report both chronic pain and pain-related disability compared to those without such histories (Bussières et al., 2023). Although the association between physical abuse and pain is particularly strong, many types of adverse experiences in childhood contribute to heightened pain in adulthood (Bussières et al., 2023; Nicolson et al., 2023). Experiencing adverse experiences such as abuse and neglect in childhood is also associated with shorter sleep duration and more self-reported sleep problems in adulthood (Vadukapuram et al., 2022). Exposure to child trauma contributes to increased risk of developing chronic fatigue symptoms in adulthood (Heim et al., 2006). Further, childhood adversity is associated with increased fatigue among college students, with symptoms of post-traumatic stress disorder serving as a potential mediator of the association (Kalmakis et al., 2022). Prospective studies also highlight that adverse childhood experiences are associated with increased odds of worse self-rated health in adulthood (Jahn et al., 2021).

Undoubtedly, child abuse can have profound and lasting effects on individuals well into adulthood, particularly for those living in rural areas, where access to mental health services and support networks may be limited. Risk factors of child maltreatment include child poverty, single-parent families, parental unemployment, neighborhood disorder, and inadequate housing, all of which are commonly present in rural areas (Smith & Pressley, 2019; National Center for Education Statistics, 2023). Children and adolescents living in rural areas may be more likely than those in urban communities to experience and report adverse childhood experiences (Maguire-Jack & Kim, 2021; Sedlak et al., 2010; Smith & Pressley, 2019). Indeed, in a study of 103,203 individuals, 56.5% of respondents living in rural areas, including the Deep South of the United States, reported exposure to at least one adverse childhood experience and 14.6% reported exposure to four or more adverse childhood experiences (Talbot et al., 2016). In rural communities, the effects of early traumatic experiences may be compounded by social isolation, stigma surrounding mental health, and a lack of specialized care, thus reducing opportunities for resilience as individuals age (Copeland et al., 2018; Hub, 2024; Talbot et al., 2016). Despite evidence suggesting that child maltreatment is higher in rural areas compared to urban areas, little work has studied the psychological and physical impact of child maltreatment in rural areas.

### Current Study

This study assessed the associations between histories of abuse and neglect and physical symptoms among young adults living in the Southeastern region of the United States. The Southeastern United States, often referred to as the Deep South, is a region of the United States that has historically limited access to both physical and mental health resources (Hub, 2024; Morales et al., 2020), subsequently heightening risk for psychological and physical health conditions. Specifically, the Deep South most commonly comprises Alabama, Mississippi, Louisiana, Georgia, and South Carolina (C. E. Miller & Vasan, 2021; Reed, 1986) and individuals living in this region often experience persistent structural barriers to healthcare access and poorer health outcomes compared to national averages (C. E. Miller & Vasan, 2021). Consistent with current literature, we hypothesized that higher rates of abuse and neglect in childhood would be associated with higher pain and fatigue. We also hypothesized that abuse and neglect would correspond to lower ratings of self-rated health and worse sleep quality. Given that early traumatic experiences can also increase depression and anxiety in adults, an additional aim of this study was to assess the mediating role of depressive and anxiety symptoms in the association between abuse/neglect and physical symptoms (i.e., pain, fatigue, self-rated health, and sleep quality). Given established direct associations (Bussières et al., 2023; Heim et al., 2006; Nicolson et al., 2023; Vadukapuram et al., 2022), we hypothesized that depressive and anxiety symptoms would partially, rather than fully, mediate the relationships between adverse childhood experiences and physical health symptoms.

## 2. Materials and Methods

### 2.1. Participants and Procedures

Participants (N = 606) were adults who took part in the study as part of research participation in their psychology courses at a large, public university in the southeastern United States. Participants had to be at least 18 years old, be able to read and understand English, and be willing to complete questionnaires related to their potential experiences of abuse and neglect prior to the age of 18.

This study was approved by the University of Southern Mississippi’s Institutional Review Board. All participants provided written informed consent prior to participation. Individuals saw the study advertised through the SONA system at the university, which was the sole recruitment method for the current study. The SONA system is a web-based participant management platform used to recruit, schedule, and manage research participants and is commonly used in university settings. All participants self-selected into the study by signing up via an online link. This link led them to an informed consent page where potential participants were able to read about the study. They completed all surveys for the study online via the Qualtrics online platform. They were compensated with 1 research credit for their time and participation. The study took approximately 30–45 min to complete.

### 2.2. Measures

Predictor Variable. The Childhood Trauma Questionnaire (CTQ) is a 28-item retrospective self-report instrument designed to assess exposure to childhood maltreatment occurring before age 18 (Bernstein et al., 1994). Respondents indicate the extent to which each statement reflects their experiences using a 5-point Likert-type scale ranging from *never true* to *very often true*. Prior work has supported the psychometric strength of the measure, demonstrating robust internal consistency across varied populations (Viola et al., 2016) (Cronbach’s α = 0.89). Evidence also supports the measure’s temporal stability, with strong test–retest reliability (r = 0.79), indicating that retrospective reports remain relatively stable across adulthood (Hardt & Rutter, 2004). For the present analyses, continuous scores from the five CTQ domains—emotional abuse, physical abuse, sexual abuse, emotional neglect, and physical neglect—were examined.

Mediating Variables. The Center for Epidemiologic Studies Depression Scale (CES-D) provided data on depressive symptoms (Radloff, 1977). The CES-D is a 20-item scale that asks participants to indicate the frequency at which they experienced depressive symptoms over the past week. Scores on the CES-D range from 0–60 with higher scores indicating greater depressive symptoms. The CES-D showed good consistency in this study (Cronbach’s α = 0.782). The 20-item State Trait Anxiety Inventory, Trait Version is a 20-item scale that asks participants to report the extent to which they experienced symptoms associated with anxiety over the past two weeks (Bieling et al., 1998). The State Trait Anxiety Inventory showed excellent internal consistency in this study (Cronbach’s α = 0.885).

Outcome Variables. Short Form 36 pain and general health subscales. The Short Form 36 (SF-36) is a 36-item self-report measure that assesses general health, well-being, and quality of life in the past 4 weeks (Hays & Morales, 2001). Participants in this study completed the pain and general health subscales. Higher scores on these scales indicate better health, well-being, and quality of life. The Multidimensional Fatigue Symptom Inventory—Short Form (MFSI-SF) is a 30-item self-report measure assessing several aspects of fatigue (Stein et al., 1998). Participants rate statements about how they have been feeling over the past 7 days. Higher scores indicate worse fatigue. The MFSI-SF showed acceptable internal consistency in this study (Cronbach’s α = 0.706). The Pittsburgh Sleep Quality Inventory (PSQI) is a 19-item self-assessment of sleep quality over the past month (Buysse et al., 1989). The PSQI yields seven component scores which are used to compile an overall sleep quality score (Buysse et al., 1989). Although the full PSQI also includes questions asking about partner sleep, only the self-report questions were administered in this study. The PSQI demonstrated good internal consistency in the current sample (Cronbach’s α = 0.817).

### 2.3. Data Analysis Plan

All analyses were conducted in SPSS version 29. Bivariate correlations between study variables examined associations among each subscale of the CTQ, depressive symptoms, anxiety symptoms, and physical symptoms. The Hayes PROCESS Macro, Model 4 (Hayes, 2012) tested simple indirect effects of childhood abuse and neglect on physical and cognitive symptoms through depressive and anxiety symptoms among participants. Indirect effects were tested with 95% bias-corrected confidence intervals, and the confidence intervals for the indirect effects were also tested with 10,000 bootstrapped samples. Mediation analyses controlled for presence of chronic medical conditions, age, race, and gender.

## 3. Results

### 3.1. Participant Characteristics

Participant demographics are provided in Table 1. Most participants in this study were White (62.4%) and female (83.9%). In terms of sexual orientation, most participants identified as straight (80.6%). A total of 33.6% of participants were first-generation college students and 10.5% were diagnosed with a chronic illness. While all students were enrolled in the university housed in a large town where this study took place, participants provided zip codes of where they spent the majority of their childhood and adolescence. Most participants (72.6%) reported that they grew up in a rural community.

### 3.2. Associations Between Childhood Abuse/Neglect and Physical Symptoms

Associations between and among study variables are presented in Table 2. Higher reporting of each type of abuse was positively associated with both anxiety and depressive symptoms except for the relationship between sexual abuse and depressive symptoms. The strength of the association between emotional abuse and depressive and anxiety symptoms was medium in strength (*r*s = 0.33–0.41) while the other associations were small in magnitude (*r*s = 0.07–0.17). There were also significant associations between each type of abuse and pain, fatigue, sleep quality, and self-rated health. The associations with between emotional abuse and fatigue and sleep quality were of a medium strength (*r*s = 0.33–0.38) while the rest of the associations between abuse and physical symptoms were of a small magnitude (*r*s = −0.12–−0.29).

While emotional neglect was associated with depressive symptoms (*r* = 0.17), there were no other associations between neglect and anxiety or depressive symptoms. There were also no significant associations between emotional or physical neglect and self-rated health, pain, or fatigue. While emotional neglect was positively associated but of a small magnitude with sleep quality (*r* = 0.21), the relationship between sleep quality and physical neglect was small and not statistically significant.

### 3.3. Mediating Effects of Depressive Symptoms

Mediating effects of depressive symptoms can be found in Table 3. Depressive symptoms mediated the association between both emotional abuse and physical abuse and pain, fatigue, sleep quality, and self-rated health. In contrast, there were no mediating effects of depressive symptoms on the association between sexual abuse, physical neglect, or emotional neglect with any outcomes.

When depressive symptoms were entered into the models as a mediating variable, the relationship between emotional abuse with self-rated health (b = −1.14, SE = 0.28, *p* < 0.001), fatigue (b = 0.04, SE = 0.01, *p* < 0.001), sleep quality (b = 0.20, SE = 0.04, *p* < 0.001), and pain (b = −1.08, SE = 0.24, *p* < 0.001) remained significant, supporting our hypotheses that depression symptoms would partially mediate the association between abuse and physical symptoms. Physical abuse remained significantly associated with self-rated health (b = −0.86, SE = 0.42, *p* = 0.04) and pain (b = −1.08, SE = 0.37, *p* < 0.01). In contrast, the association between physical abuse and fatigue (b = 0.02, SE = 0.01, *p* = 0.09) was no longer significant with depressive symptoms entered into the models as a mediating variable. Similarly, physical abuse was no longer directly associated with sleep quality (b = 0.11, SE = 0.06, *p* = 0.09) with depressive symptoms entered into the model. Overall, these models show that depressive symptoms partially mediated the association between emotional abuse and all physical health outcomes, along with the associations between physical abuse with pain and self-rated health. In contrast, these mediation models support full mediation of depressive symptoms in the link between physical abuse fatigue and sleep quality.

### 3.4. Mediating Effects of Anxiety Symptoms

Table 4 presents the mediating effects of anxiety symptoms in the association between childhood trauma and physical symptoms. Anxiety symptoms mediated the associations between emotional abuse, physical abuse, and sexual abuse with pain, fatigue, sleep quality, and self-rated health. Like models using depressive symptoms as a mediator, there was no mediating effect of anxiety symptoms on the association between physical or emotional neglect and any outcomes.

With anxiety symptoms entered into the model, the direct effect of emotional abuse on sleep quality (b = 0.17, SE = 0.04, *p* < 0.001) remained significant, suggesting that anxiety only partially mediates the association between emotional abuse and sleep quality. The direct association between emotional abuse and self-rated health (b = −1.07, SE = 0.29, *p* < 0.01), pain (b = −1.25, SE = 0.26, *p* < 0.001) and fatigue (b = 0.03, SE = 0.01, *p* < 0.001) also remained significant with anxiety symptoms entered into the model as a mediating variable. These models show a consistent pattern of anxiety symptoms partially mediating the association between childhood abuse and physical symptoms.

Although the direct association between physical abuse and sleep quality was significant (b = 0.31, SE = 0.09, *p* < 0.001), with anxiety symptoms in the model, this direct association was no longer significant (b = 0.07, SE = 0.06, *p* = 0.20). The association between physical abuse and self-rated health was also no longer significant with anxiety symptoms entered into the model (b = −0.73, SE = 0.42, *p* = 0.08). Similarly, the association between physical abuse (b = 0.01, SE = 0.01, *p* = 0.34) with fatigue was no longer significant. In contrast, the association between physical abuse and pain remained significant with anxiety symptoms entered into the model (b = −1.11, SE = 0.39, *p* < 0.01). In summary, these models indicate that, overall, anxiety symptoms fully mediated the associations between physical abuse and sleep quality, self-rated health, and fatigue. In contrast, anxiety symptoms appeared to only partially mediate the link between physical abuse and pain.

With anxiety symptoms entered into the model, the direct effect of sexual abuse on sleep quality remained significant (b = 0.15, SE = 0.05, *p* < 0.01). The association between sexual abuse (b = −0.75, SE = 0.33, *p* = 0.02) and pain also remained significant with anxiety symptoms entered into the model as a mediating variable. In contrast, the associations with self-rated health (b= −0.62, SE = 0.37, *p* = 0.09) and fatigue (b = 0.01, SE = 0.01, *p* = 0.43) with sexual abuse were no longer significant with anxiety symptoms entered into the model as a mediating variable. Overall, anxiety symptoms partially mediated the associations between sexual abuse and pain and sleep quality, while only partially mediating sexual abuse’s links with self-rated health and fatigue.

## 4. Discussion

Addressing the long-term health effects of childhood adversity is a clear priority of the Center for Disease Control (CDC, 2024). Childhood adversity’s clear links to physical health issues in adulthood—including cardiovascular disease, diabetes, and cancer—highlight the necessity of identifying both biological and psychological mechanisms underlying this relationship (Fagundes et al., 2013; G. E. Miller et al., 2011). Accordingly, this study examined associations between childhood abuse/neglect, anxiety symptoms, depressive symptoms, and physical symptoms among young adults living in the Deep South, a region of the United States that experiences numerous mental and physical health disparities (C. E. Miller & Vasan, 2021). We hypothesized that higher depressive symptoms, anxiety symptoms, and abuse/neglect reporting would correspond to worse pain, fatigue, sleep quality, and self-rated health. Given that trauma histories can increase symptoms of anxiety and depression (Dalechek et al., 2024; Merrick et al., 2017), we also hypothesized that anxiety and depressive symptoms would mediate the association between abuse/neglect histories and physical symptoms among these rural-dwelling young adults.

Higher endorsement of each type of abuse and neglect corresponded to worse physical symptoms among the participants in this study. These associations are consistent with past research linking abuse and neglect histories to worse sleep, fatigue, self-rated health, and pain among adults (Gama et al., 2021; Norman et al., 2012; Walker et al., 1999). This study expanded these findings to specifically focus on adults living in the rural south, an area that is historically under-resourced for physical and mental healthcare. Specifically, the Deep South is a region in which many communities experience persistent socioeconomic disadvantage, shortages of healthcare providers, reduced access to preventive and specialty care, and a disproportionately high burden of chronic disease (Cyr et al., 2019; C. E. Miller & Vasan, 2021; Thomas et al., 2014). Further, consistent with study hypotheses, both depressive and anxiety symptoms mediated the association between physical abuse and emotional abuse with pain, fatigue, self-rated health, and sleep quality. Interestingly, only anxiety symptoms mediated the association between histories of sexual abuse and the physical symptoms measured in this study. Inconsistent with study hypotheses, depressive and anxiety symptoms did not mediate the associations between neglect histories (emotional or physical) and physical symptoms. These findings highlight anxiety and depressive symptoms as a potential mechanism linking early life experiences to physical health among the young adults in this study. Previous research has highlighted trauma symptoms as a potential mediator of the association between abuse in childhood and self-reported physical health and somatic symptoms among adolescents and adults (Ho et al., 2021; Rueness et al., 2019). Psychological distress, as measured by symptoms pertaining to both post-traumatic stress disorder and depression, also mediate the association between abuse and physical health among young adults (Beck et al., 2014). The current study’s findings add to this body of literature by highlighting both anxiety and depressive symptoms as potential mediators of the association between physical and emotional abuse and physical health. While prior studies have examined depression and trauma symptoms as mediators, little work has tested anxiety and its influence on the relationship between abuse histories and physical health. Further, using the CTQ allowed us to understand differing mediation effects with both anxiety and depressive symptoms, and whether these associations held for different types of abuse and neglect. Given that these associations were only shown in cross-sectional data in this study, longitudinal studies are needed to replicate these findings to establish temporal relationships among trauma, psychological symptoms, and physical health.

These findings contribute to a growing body of literature emphasizing the impact that early adverse life experiences have on physical and mental health as individuals age (Lovis-Schmidt et al., 2024; Sachs-Ericsson et al., 2005; Wegman & Stetler, 2009). Although childhood abuse and neglect confer risk for the development of chronic health conditions, even in the absence of chronic illness, these trauma histories worsen physical symptoms, which can reduce quality of life and overall functioning (Bussières et al., 2023; Heim et al., 2006; Nicolson et al., 2023; Vadukapuram et al., 2022). Consistent with the biological embedding model, this study suggests that childhood abuse has lasting negative effects on pain, sleep, self-rated health, and fatigue in adulthood, even among those without chronic conditions (Danese et al., 2011). Subsequently, our findings offer several identifiable ways to mitigate the effects of childhood abuse and neglect on health as individuals age. However, these findings also highlight important areas of policy change within communities, including enhancing mandated reporting systems, expanding early-intervention programs for children and adolescents who have experienced abuse and neglect, and supporting community-based child maltreatment prevention efforts. Indeed, enhancing policies around the reporting and intervention on childhood abuse and neglect may help to break the link between these experiences and long-term physical health symptoms and conditions.

The participants in this study were living within the Deep South, a region of the United States with limited access to both physical and mental health resources (Hub, 2024; Morales et al., 2020). Indeed, over 60% of Americans living in rural areas live in shortage areas for mental health providers, highlighting a critical need for specialized mental health care in these areas (Kepley & Streeter, 2018). Despite research suggesting that people living in rural areas, including those in the southern United States, may be more likely to experience abuse and trauma compared to those in more urban communities, there continues to be a lack of research on the impact of such experiences for those living in rural areas (Maguire-Jack & Kim, 2021; Sedlak et al., 2010; Smith & Pressley, 2019). Results from this study underscore the need for continued research on and interventions for individuals living in rural areas with histories of abuse and neglect in their childhood. More broadly, these results underscore the need for trauma-informed public health initiatives with a specific emphasis on expanding these practices in communities within the Deep South. While the participants in this study were college students, thus giving them access to mental and physical healthcare through the university system, we did not collect data on what percentage of the sample took advantage of these services. Results also highlight several notable avenues for university-related changes to better address the intersection between physical and mental health among college students. Importantly, our findings suggest the importance of college counseling centers and health clinics having trauma-informed trainings to better address the physical and mental health consequences of childhood abuse and neglect. University health centers may also benefit from implementing routine mental health screenings if not already in place to better understand the psychological symptoms that may be contributing to or coinciding with physical health complaints.

This study used several different health outcomes that may or may not be associated with chronic illnesses. The use of multiple outcomes allows for a broad-based understanding of the association between abuse and neglect histories and physical symptoms. Further, the use of the CTQ allowed us to examine different types of abuse and neglect and their associations with physical health. Importantly, emotional abuse, emotional neglect, and physical neglect are not considered Criterion A events based on DSM-5 criteria for post-traumatic stress disorder (American Psychiatric Association, 2013). Our findings show that a broader conceptualization of traumatic experiences beyond the DSM-5 established criteria may have lasting impacts on both physical and mental health. Although the majority of participants in this sample identified racially as white, nearly 30% identified as black or African American. This percentage is largely reflective of the racial makeup of the region where the study took place (U.S. Census Bureau, 2023), thus providing a relatively representative subsample of the population. Despite these strengths, this study is not without limitations that warrant consideration. The cross-sectional nature of this study limits causal inferences in our models, making it difficult to determine anxiety and depressive symptoms as true mechanisms underlying the association between abuse/neglect and self-reported physical symptoms. The age of participants in this study was relatively young on average, and results should be replicated with adults in middle and older age to enhance generalizability across adulthood. Although the CTQ assesses several types of abuse and neglect, this study did not assess other potentially traumatic experiences in childhood and adolescence. Future research should replicate findings from this study using a broader assessment and conceptualization of trauma to better understand its impact on health. While most participants in our sample reported growing up in rural communities based on the zip codes they provided during study participation, all of them were attending the same university in a large town within the Deep South. While we did not obtain data on the frequency of its use, all participants had access to low-cost medical care at the on-campus health clinic and free counseling services. Thus, while our participants were living in, and, for the majority of them, had been raised within the Deep South, this sample may not be generalizable to community participants without this type of healthcare access and educational attainment. Lastly, the CTQ uses retrospective reports of abuse. Prospective and retrospective abuse reports generally have low agreement with one another (Baldwin et al., 2019). Further, prospective reports of abuse previously correlated more strongly with objective and subjective physical health reporting compared to retrospective abuse reports (Reuben et al., 2016).

This study highlights how anxiety and depressive symptoms may heighten risk for poorer self-reported physical health in adults who experienced abuse earlier in life. These findings highlight the necessity of policies focused on training providers in and practicing trauma-informed care across the lifespan and screening for abuse/neglect to try to provide early intervention to those exposed to such adverse experiences in childhood and adolescence. Future research should focus on examining these associations longitudinally to establish causality in childhood abuse and neglect worsening psychological and physical health. Interventions targeting psychological symptoms associated with childhood abuse may therefore improve both the psychological and physical health of adults as they age.

## Figures and Tables

**Table 1:** Participant Demographics.

	M	(SD)	N	%
Age	20.90	5.82		
Gender				
*Male*			83	13.7%
*Female*			510	83.9%
*Non-binary*			12	2.0%
*Transgender*			1	0.2%
Race				
*White*			378	62.4%
*Black*			180	29.7%
*Asian American*			26	4.3%
*Pacific Islander*			1	0.2%
*Native American/Alaskan Native*			2	0.3%
*Mixed Race*			19	3.1%
Sexual Orientation				
*Straight*			487	80.6%
*Gay*			18	3.0%
*Bisexual*			65	10.7%
*Asexual*			6	1.0%
*Pansexual*			13	2.1%
*Other*			4	0.7%
*Prefer not to say*			11	1.8%
First-Generation			204	33.6%
Rural Upbringing			440	72.6%
Chronic Illness			64	10.5%

Note. M = mean; SD = standard deviation; N = number of participants.

**Table 2:** Correlations between and among variables.

	1	2	3	4	5	6	7	8	9	10	11
1. Emotional Abuse	-										
2. Physical Abuse	0.45 **	-									
3. Sexual Abuse	0.35 **	0.37 **	-								
4. Emotional Neglect	0.18 *	0.22 **	0.12	-							
5. Physical Neglect	0.22 *	0.27 **	0.35 **	0.38 **	-						
6. Depression	0.33 **	0.11 *	0.07	0.17 *	0.02	-					
7. Anxiety	0.41 **	0.15 **	0.11 **	0.07	0.05	0.56 **	-				
8. Self-Rated Health	−0.27 **	−0.12 **	−0.11 *	−0.02	−0.05	−0.31 **	−0.34 **	-			
9. Pain	−0.29 **	−0.16 **	−0.13 **	−0.08	−0.10	−0.38 **	−0.27 **	0.46 **	-		
10. Fatigue	0.38 **	0.13 **	0.10 *	0.10	0.05	0.66 **	0.65 **	−0.46 **	−0.55 **	-	
11. Sleep Quality	0.33 **	0.12 **	0.16 **	0.21 **	0.07	0.47 **	0.50 **	−0.37 **	−0.38 **	0.54 **	-

Note. Emotional abuse, physical abuse, sexual abuse, emotional neglect, and physical neglect were measured using the Childhood Trauma Questionnaire. Depressive symptoms were measured using the Center for Epidemiological Studies—Depression Scale. The State Trait Anxiety Inventory, Trait version provided information on anxiety symptoms. The Short Form-36’s pain and general health subscales measured pain and self-rated health. Fatigue data was captured using the Multidimensional Fatigue Symptom Inventory, short form. Sleep quality was measured using the Pittsburgh Sleep Quality Inventory. * *p* < 0.05, ** *p* < 0.01.

**Table 3:** Mediating effects of depressive symptoms.

	Indirect Effects		
	*b*	*SE*	95% CI
Physical Abuse (*X*_1_)			
Pain (*Y*_1_)	−0.35	0.17	**−0.70, −0.03**
Self-Rated Health (*Y*_2_)	−0.31	0.15	**−0.63, −0.03**
Fatigue (*Y*_3_)	0.03	0.02	**0.03, 0.05**
Sleep Quality (*Y*_4_)	0.08	0.04	**0.01, 0.15**
Emotional Abuse (*X*_2_)			
Pain (*Y*_1_)	−0.47	0.11	**−0.71, −0.28**
Self-Rated Health (*Y*_2_)	−0.49	0.13	**−0.77, −0.27**
Fatigue (*Y*_3_)	0.04	0.01	**0.03, 0.05**
Sleep Quality (*Y*_4_)	0.12	0.02	**0.08, 0.17**
Sexual Abuse (X_3_)			
Pain (*Y*_1_)	−0.17	0.14	−0.46, 0.09
Self-Rated Health (*Y*_2_)	−0.17	0.14	−0.48, 0.09
Fatigue (*Y*_3_)	0.01	0.01	−0.01, 0.03
Sleep Quality (*Y*_4_)	0.04	0.03	−0.02, 0.10
Physical Neglect (X_4_)			
Pain (*Y*_1_)	−0.03	0.16	−0.34, 0.32
Self-Rated Health (*Y*_2_)	−0.02	0.13	−0.29, 0.25
Fatigue (*Y*_3_)	0.003	0.01	−0.03, 0.03
Sleep Quality (*Y*_4_)	0.01	0.05	−0.09, 0.10
Emotional Neglect (X_5_)			
Pain (*Y*_1_)	−0.30	0.17	−0.63, 0.03
Self-Rated Health (*Y*_2_)	−0.21	0.13	−0.50, 0.04
Fatigue (*Y*_3_)	0.02	0.01	−0.002, 0.05
Sleep Quality (*Y*_4_)	0.08	0.04	−0.01, 0.16

Note. SE = standard error; 95% CI = 95% confidence interval; b = beta. Emotional abuse, physical abuse, sexual abuse, emotional neglect, and physical neglect were measured using the Childhood Trauma Questionnaire. Depressive symptoms were measured using the Center for Epidemiological Studies—Depression Scale. The Short Form-36’s pain and general health subscales measured pain and self-rated health. Fatigue data was captured using the Multidimensional Fatigue Symptom Inventory, short form. Sleep quality was measured using the Pittsburgh Sleep Quality Inventory. Bold font indicates significant mediating effects.

**Table 4:** Mediating effects of anxiety symptoms.

	Indirect Effects		
	*b*	*SE*	95% CI
Physical Abuse (*X*_1_)			
Pain (*Y*_1_)	−0.33	0.11	**−0.58, −0.13**
Self-Rated Health (*Y*_2_)	−0.48	0.15	**−0.80, −0.21**
Fatigue (*Y*_3_)	0.03	0.01	**0.01, 0.05**
Sleep Quality (*Y*_4_)	0.11	0.03	**0.05, 0.18**
Emotional Abuse (*X*_2_)			
Pain (*Y*_1_)	−0.32	0.11	**−0.54, −0.11**
Self-Rated Health (*Y*_2_)	−0.57	0.14	**−0.86, −0.30**
Fatigue (*Y*_3_)	0.05	0.01	**0.04, 0.06**
Sleep Quality (*Y*_4_)	0.16	0.02	**0.11, 0.21**
Sexual Abuse (X_3_)			
Pain (*Y*_1_)	−0.22	0.09	**−0.41, −0.07**
Self-Rated Health (*Y*_2_)	−0.36	0.13	**−0.64, −0.12**
Fatigue (*Y*_3_)	0.02	0.01	**0.01, 0.04**
Sleep Quality (*Y*_4_)	0.07	0.03	**0.03, 0.13**
Physical Neglect (X_4_)			
Pain (*Y*_1_)	−0.07	0.15	−0.36, 0.25
Self-Rated Health (*Y*_2_)	−0.07	0.16	−0.41, 0.24
Fatigue (*Y*_3_)	0.01	0.02	−0.02, 0.04
Sleep Quality (*Y*_4_)	0.03	0.05	−0.07, 0.14
Emotional Neglect (X_5_)			
Pain (*Y*_1_)	−0.09	0.11	−0.31, 0.13
Self-Rated Health (*Y*_2_)	−0.10	0.13	−0.38, 0.14
Fatigue (*Y*_3_)	0.01	0.01	−0.01, 0.03
Sleep Quality (*Y*_4_)	0.04	0.04	−0.05, 0.12

Note. SE = standard error; 95% CI = 95% confidence interval; b = beta. Emotional abuse, physical abuse, sexual abuse, emotional neglect, and physical neglect were measured using the Childhood Trauma Questionnaire. The State Trait Anxiety Inventory, Trait version provided information on anxiety symptoms. The Short Form-36’s pain and general health subscales measured pain and self-rated health. Fatigue data was captured using the Multidimensional Fatigue Symptom Inventory, short form. Sleep quality was measured using the Pittsburgh Sleep Quality Inventory. Bold font indicates significant mediating effects.

## Data Availability

Data is available upon request from the corresponding author.
